# Baroreflex responses during dry resting and exercise apnoeas in air and pure oxygen

**DOI:** 10.1007/s00421-020-04544-w

**Published:** 2020-11-05

**Authors:** Anna Taboni, Giovanni Vinetti, Timothée Fontolliet, Gabriele Simone Grasso, Enrico Tam, Christian Moia, Guido Ferretti, Nazzareno Fagoni

**Affiliations:** 1 Department of Anaesthesiology, Pharmacology, Intensive Care, and Emergencies, University of Geneva, 1 rue Michel-Servet, CH-1211 Genève 4, Switzerland; 2 Department of Molecular and Translational Medicine, University of Brescia, Brescia, Italy; 3 School of Medicine and Surgery, University of Milano, Bicocca, Monza, Milano, Italy; 4 Department of Neuroscience, Biomedicine and Movement, University of Verona, Verona, Italy; 5 AAT Brescia, Agenzia Regionale Emergenza Urgenza (AREU), ASST Spedali Civili di Brescia, Brescia, Italy

**Keywords:** Baroreflex resetting, Baroreflex sensitivity, Breath holding, Closed loop, Sequence method

## Abstract

**Purpose:**

We analysed the characteristics of arterial baroreflexes during the first phase of apnoea (φ1).

**Methods:**

12 divers performed rest and exercise (30 W) apnoeas (air and oxygen). We measured beat-by-beat R-to-R interval (RRi) and mean arterial pressure (MAP). Mean RRi and MAP values defined the operating point (OP) before (PRE-ss) and in the second phase (φ2) of apnoea. Baroreflex sensitivity (BRS, ms·mmHg^−1^) was calculated with the sequence method.

**Results:**

In PRE-ss, BRS was (median [IQR]): at rest, 20.3 [10.0–28.6] in air and 18.8 [13.8–25.2] in O_2_; at exercise 9.2[8.4–13.2] in air and 10.1[8.4–13.6] in O_2_. In φ1, during MAP decrease, BRS was lower than in PRE-ss at rest (6.6 [5.3–11.4] in air and 7.7 [4.9–14.3] in O_2_, *p* < 0.05). At exercise, BRS in φ1 was 6.4 [3.9–13.1] in air and 6.7 [4.1–9.5] in O_2_. After attainment of minimum MAP (MAPmin), baroreflex resetting started. After attainment of minimum RRi, baroreflex sequences reappeared. In φ2, BRS at rest was 12.1 [9.6–16.2] in air, 12.9 [9.2–15.8] in O_2_. At exercise (no φ2 in air), it was 7.9 [5.4–10.7] in O_2_. In φ2, OP acts at higher MAP values.

**Conclusion:**

In apnoea φ1, there is a sudden correction of MAP fall via baroreflex. The lower BRS in the earliest φ1 suggests a possible parasympathetic mechanism underpinning this reduction. After MAPmin, baroreflex resets, displacing its OP at higher MAP level; thus, resetting may not be due to central command. After resetting, restoration of BRS suggests re-establishment of vagal drive.

## Introduction

The time course of cardiovascular parameters during dry apnoeas has been investigated at rest and exercise, in air and after breathing pure oxygen (Perini et al. 2008; Tocco et al. 2012; Costalat et al. 2013, 2015; Sivieri et al. 2015; Fagoni et al. 2015, 2017; Taboni et al. 2018b, 2019). All these studies show that, at the beginning of resting dry apnoeas, carried out after a deep inspiration, a sudden fall in mean arterial pressure (MAP) is corrected by an increase in heart rate (HR) (phase 1, φ1). After attainment of a minimum MAP value (MAPmin), both MAP and HR recover to attain a new level, close to the resting value, which is maintained stable for more than one minute (phase 2, φ2).

When the apnoeas are performed at lung volumes close to the individual total lung capacity, there might be a reduction of venous return during φ1, due to the high intrathoracic pressure exerted at elevated lung volumes, which entails an increase in central venous pressure and a decrease in cardiac output and in MAP (Potkin et al. 2007; Breskovic et al. 2011a; Batinic et al. 2011; Stembridge et al. 2017). These cardiovascular changes would be similar to those that intervene in the course of a Valsalva manoeuvre (Korner et al. 1976; Palmero et al. 1981). Therefore, φ1 was interpreted as the baroreflex attempt to correct the reduction in MAP (Fagoni et al. 2015). Bringard et al. (2017) observed similar patterns in the first seconds of light exercise (Bringard et al. 2017). They demonstrated that, before the opposite variations of R-to-R interval (RRi) and MAP leading to the arterial baroreflex resetting at exercise, there is an initial progressive consensual decrease of RRi and MAP along a specific baroreflex curve. Thus, an analysis of the baroreflex dynamics during apnoea could shed light on the characteristics of the baroreflex response to MAP perturbations at the beginning of breath-holding.

We hypothesised that during φ1, both in resting and exercise apnoeas, MAP and HR follow patterns that are similar to those occurring at exercise start, including the occurrence of arterial baroreflex resetting. Furthermore, during exercise apnoeas the characteristics of φ1 would be different from resting apnoeas, because the baroreflex sensitivity (BRS) measured at exercise determined at steady state before apnoea start is lower than at rest, and the operating point of the baroreflex is located downward and rightward in the RRi versus MAP plot (Iellamo et al. 1997; Vallais et al. 2009; Fontolliet et al. 2018). A test of these hypotheses requires an analysis of the characteristics of the cardiac-chronotropic component of arterial baroreflex responses during breath-holding. Since φ1 is an unsteady state condition, only closed-loop methods can conveniently be used for the analysis of arterial baroreflexes (Adami et al. 2013; Bringard et al. 2017). Therefore, to test these hypotheses, we performed a closed-loop analysis of the arterial baroreflexes during φ1 and φ2 that characterise the cardiovascular time course of a dry apnoea performed at high lung volumes. Apnoeas were carried out at lung volumes close to the total lung capacity, at rest and during exercise, in air and during pure oxygen administration.

## Matherials and methods

### Subjects

The experiments were carried out at sea level, during an international dynamic apnoea competition in Lignano Sabbiadoro (Italy).

Overall, 12 divers volunteered for this study, ten males and two females. They were 40 ± 8 years old, 177 ± 6 cm tall, and 76 ± 9 kg heavy (anthropometric data are expressed as mean ± standard deviation, SD). None of them had previous history of cardiovascular, pulmonary, or neurological diseases, nor was taking medications at the time of the study. All gave their informed consent after having received a detailed description of the methods and experimental procedures of the study. The study conformed to the Declaration of Helsinki and was approved by the local ethical committee.

### Experimental procedure

Subjects came to the laboratories on two occasions: one for the tests in air and one for the tests in oxygen. Upon arrival to the laboratory the subjects took the supine position on a table and, after instrumentation, they were familiarized with procedures. After 300 s of quiet rest, the protocol started by recording data during further 300 s at rest, to obtain pre apnoea steady-state values (PRE-ss). Then, the subjects performed a series of five sub-maximal apnoeas of at least 30 s plus one maximal apnoea, separated by a recovery interval of 120 s. At the end of the last apnoea at rest and after a short recovery, the protocol at exercise took place, which was performed as follows. A cycle-ergometer (Ergoselect 400, Ergoline GmbH, Bitz, Germany) was mounted on the table, then in supine position the subject started to pedal at 30 W, 60 rpm, and kept exercising until the end of the procedure. Parameters recording started after attainment of the exercise steady state (Ferretti et al. 2017). Then, after further 300 s, to obtain PRE-ss values, the subjects performed a series of five sub-maximal apnoeas of at least 30 s plus one maximal apnoea.

On the day of the tests in oxygen, the experiments were carried out while breathing pure oxygen, which was delivered by means of a two-way non-rebreathing T-shape valve (Hans Rudolph, Inc., Shawnee, KS, USA). The inhalation port was connected to a 200 l Douglas bag, used as pressure buffer system, filled with 100% oxygen coming from a high-pressure tank. After connection, 10 min of quiet breathing were allowed to attain alveolar gas equilibration (Darling et al. 1940) before performing the procedure.

In all cases, subjects undertook their pre-dive routine breathing before breath-holding, generally consisting of a couple of deep respiratory acts, ending with a deep inspiration, so that the lung volume at which the apnoeas started was close to the subject’s total lung capacity.

### Measurements and data treatment

In apnoea φ1, which is a dynamic unsteady state condition, only closed-loop method can be employed to analyse baroreflex responses (Bringard et al. 2017). Sequences of consecutive beats in which RRi and MAP increase or decrease concurrently can be identified (Sivieri et al. 2015; Fagoni et al. 2015), defining linear segments of the RRi versus MAP relationship (Taboni et al. 2018a). As long as RRi and MAP vary consensually, their slope has the same meaning as the closed-loop BRS calculated at steady state by means of the sequence method (Bertinieri et al. 1988; Parati et al. 2000).

With this concept in mind, we obtained continuous recordings of arterial blood pressure profiles (Portapres^®^, Finapres^®^ Medical Systems BV, Enschede, The Netherlands) and electrocardiogram (ECG100C module, BIOPAC^®^ Systems Inc., Goleta, CA, USA) throughout the entire protocol. Peripheral blood O_2_ saturation was measured at an earlobe (infrared spectroscopy, Nellcor™ N-595, Nellcor Puritan Bennet Inc., Pleasanton, CA, USA) for safety during maximal apnoeas (Vinetti et al. 2020). Blood pressure was also measured at rest by means of a sphygmomanometer placed on the contralateral arm, to monitor the reliability of pressure measure obtained by the Portapres^®^. A difference in MAP > 10 mmHg between the two methods, implied recalibration of the Portapres^®^ and repetition of the protocol. The signals were sampled at 400 Hz using a 16-bit A/D converter (MP150, BIOPAC^®^ Systems Inc., Goleta, CA, USA) and stored on a personal computer for subsequent analysis. The breath-by-breath recording of inspiratory and expiratory flows was performed by an ultrasonic flowmeter (Spiroson^®^, ECO MEDICS AG, Duernten, Switzerland) calibrated with a 3 l syringe. Flat flow signals provided the beginning, ending, and duration of apnoeas.

The time series of RRi during the entire recording were computed from the ECG traces on a beat-by-beat basis. Arterial pressure profiles were analysed offline, using the Beatscope^®^ software (Finapres^®^ Medical Systems BV, Enschede, The Netherlands), to obtain beat-by-beat values of systolic (SAP), diastolic arterial pressure, and MAP.

Maximal apnoeas were analysed to identify the three phases of apnoeas, both in air and in oxygen, while the sub-maximal apnoeas were analysed to identify φ1 and the beginning of φ2, and to compute φ1 duration (Sivieri et al. 2015; Fagoni et al. 2015, 2017, 2018; Breenfeldt Andersen et al. 2018). The PRE-ss HR and MAP and their respective values and during φ2 were computed from the maximal apnoeas, both at rest and during exercise. Only exception concerned exercise apnoeas in air, for the lack of φ2 (Sivieri et al. 2015; Taboni et al. 2018b, 2020). An automated procedure implemented under MATLAB (version 7.6.0.324, MathWorks^®^, Natick, MA, USA) was used to this aim. This procedure is based on linear regression analysis, allowing detection of changes in slope between successive phases (Sivieri et al. 2015; Fagoni et al. 2015, 2017, 2018).

The BRS during quiet breathing at rest and during φ2 was calculated with the sequence method (Bertinieri et al. 1988), using MAP and RRi as independent and dependent variable, respectively. Briefly, after introducing a phase shift of 1 beat between MAP and RRi, sequences of three or more consecutive beats characterised by consensual increase or decrease in MAP and RRi were identified. Within each sequence, the relationship between RRi and MAP was analysed by linear regression to compute the slope and the coefficient of determination (*R*^2^). Only slopes with *R*^2^ > 0.85 were retained (Iellamo et al. 1997). For each subject, the mean slope of the RRi versus MAP relationships was computed and taken as a measure of individual BRS in every steady state condition. The operating point of each steady state condition, pre-apnoea and φ2, both at rest and at exercise, was computed as the average of RRi and MAP value.

A similar approach was applied to the analysis of the BRS in φ1, in analogy with what was done to investigate baroreflex dynamics at exercise onset (Bringard et al. 2017). During this phase, the RRi versus MAP relationship was established, and the single-beat minimum of MAP was identified (MAPmin) (Fagoni et al. 2015). This allowed division of φ1 in two distinct parts, characterised by continuous fall and continuous increase in MAP, respectively. Baroreflex sequences were then identified on each part of φ1.

### Statistical analysis

Data were tested for normality by means of Shapiro–Wilk test. Overall, the data relating to BRS slopes were not normally distributed, whereas OP data were normally distributed. Hence, the former were presented as median and interquartile range (IQR), and the latter as mean and SD.

Data non-normally distributed were analysed by means of the Friedman’s test and the Kruskal–Wallis’ test for paired and non-paired data, respectively. In both cases, the Dunn multiple comparison post hoc analysis was performed to locate differences.

The one-way ANOVA with Tukey post hoc test for normally distributed data (OP, MAP and RRi analysis).

Differences were considered significant when *p* < 0.05, otherwise they were considered non-significant (NS). The Prism 7.0 statistical software (GraphPad Software LLC, San Diego, CA, USA) has been used to this purpose.

## Results

All the maximal apnoeas are characterised by the well-established three phases, except the apnoeas carried out in air during exercise, in which φ2 was not observed. Thus, all the apnoeas were included in the analysis of φ1.

In resting steady state before apnoea (PRE-rest) in air, RRi was 924 ± 130 ms (corresponding to a HR of 65 beats·min^−1^) and MAP was 82 ± 10 mmHg; in exercise steady state before apnoea (PRE-ex) in air, RRi was 687 ± 69 ms (corresponding to a HR of 87 beats·min^−1^) and MAP was 91 ± 11 mmHg. In oxygen, RRi was 916 ± 156 ms (corresponding to a HR of 66 beats·min^−1^) and MAP was 84 ± 16 mmHg in PRE-rest; the corresponding values in PRE-ex were: RRi 720 ± 67 ms (corresponding to a HR of 83 beats·min^−1^) and MAP 99 ± 11 mmHg. The RRi values at exercise were significantly lower than at rest, either in air or in oxygen; MAP values were higher during exercise apnoeas in oxygen (*p* < 0.05) and in air the trend was similar but did not reach the statistical significance.

In air at rest, φ2 RRi was 847 ± 189 ms (corresponding to a HR of 71 beats·min^−1^) and φ2 MAP was 98 ± 13 mmHg (*p* < 0.05 vs. corresponding PRE-rest value); during φ2 in oxygen, RRi was 817 ± 217 ms (corresponding to a HR of 73 beats·min^−1^) and MAP was 100 ± 18 mmHg (*p* < 0.05 vs corresponding PRE-rest value). During φ2 in oxygen at exercise, RRi was 683 ± 83 ms (corresponding to a HR of 88 beats·min^−1^) and MAP was 104 ± 14 mmHg. The RRi values in φ2 did not differ from those in PRE-ex. MAP in φ2 was significantly higher than in PRE-rest, both in air and in oxygen, but no difference was found compared to PRE-ex in oxygen.

**Fig. 1:**
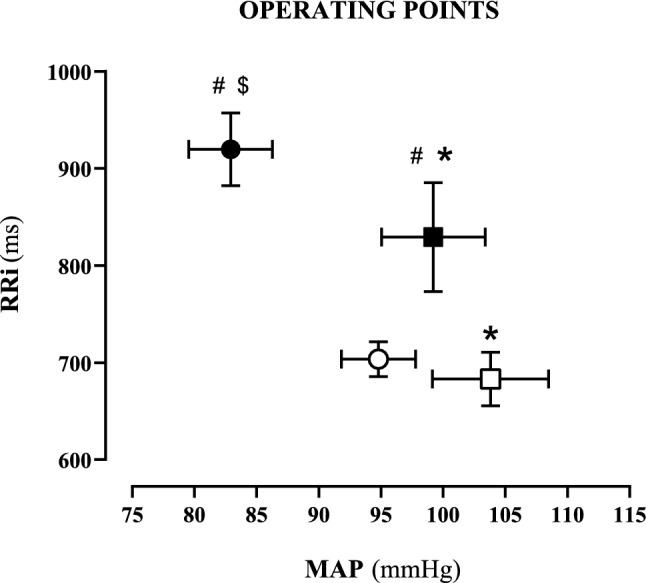
Operating points during the steady states, in all resting and exercise apnoeas. Data are presented as means and standard error of the mean. Steady state during resting (black symbols) and exercise (white symbols) apnoeas. Steady state before starting apnoeas (dots) and during φ2 (squares). RRi: R-to-R interval; MAP: mean arterial pressure; *: MAP significantly different (*p* < 0.05) than corresponding value at pre-apnoea steady state; ^#^: RRi significantly different (*p* < 0.05) than corresponding value at rest; ^$^: MAP significantly different (*p* < 0.05) than corresponding value at exercise

Table 1 reports the BRS values in air and in oxygen apnoeas. No differences between air and oxygen apnoeas were observed. This allowed construction of Fig. 1, in which data in air and oxygen were pooled together to analyse the OP in steady state conditions.

**Table 1:** Baroreflex sensitivity during steady states for resting and exercise apnoeas, in air and in oxygen

Baroreflex sensitivity during steady state (ms·mmHg^−1^)	PRE-ss	φ2
Resting apnoeas		
Air		
Median	20.3	12.1
25°–75° centile	10.0–28.6	9.6–16.2
*n*. of observations	12	12
Oxygen		
Median	18.8	12.9^#^
25°–75° centile	13.8–25.2	9.2–15.8
*n*. of observations	12	12
Exercise apnoeas		
Air		
Median	9.2	n/a
25°–75° centile	8.4–13.2	
*n*. of observations	12	
Oxygen		
Median	10.1*	7.9*^$^
25°–75° centile	8.4–13.6	5.4–10.7
*n*. of observations	12	8

*PRE-ss* steady state before apnoea

*p < 0.05 compared to corresponding value at rest

^#^*p* < 0.05 compared to PRE-ss

^$^*p* < 0.05 compared to PRE-ss in exercise air and oxygen apnoeas

OP was displaced downward in exercise compared to rest (white dot, Fig. 1), due to lower RRi (higher HR). In resting apnoeas, OP was displaced rightward, due to higher MAP in in φ2 (99.2 ± 14.5 mmHg) than in PRE-rest (82.9 ± 11.6 mmHg, *p* < 0.05), while RRi was similar. The same trend was found during oxygen exercise apnoeas, MAP φ2 was 103.8 ± 13.9 mmHg and MAP PRE-ex was 94.8 ± 10.3 mmHg (*p* < 0.05).

Several repetitions of φ1 have been obtained from the sub-maximal apnoeas. The time course of MAP during φ1 was the same in all cases, with an initial MAP decrease until the attainment of MAPmin, followed by a progressive increase of MAP, which lasted until the beginning of φ2. In air, MAPmin was 67 ± 12 mmHg at rest and 72 ± 13 mmHg at exercise, while in oxygen, MAPmin was 67 ± 12 mmHg at rest and 83 ± 17 mmHg at exercise. MAPmin resulted higher at exercise than at rest, both in air and in oxygen apnoeas.

**Fig. 2:**
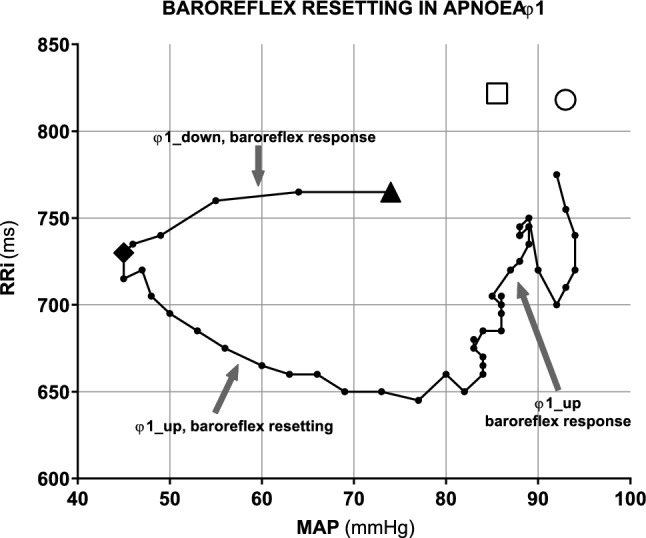
Closed-loop contour plot of the relationship between R-to-R interval (RRi) and mean arterial pressure (MAP) during φ1 in resting apnoea. Data obtained from a representative subject, showing the presence of a baroreflex responses before the attainment of minimum of mean arterial pressure (MAPmin, black diamond). The resetting phase starts from the diamond point: during this phase, RRi and MAP vary in opposite directions toward a new operating point in φ2 (white circle). White square: operating point at steady state before apnoea. Black tringle: beginning of apnoeas

An example of a contour plot representing the evolution of single-beat RRi as a function of single-beat MAP in φ1 is reported in Fig. 2.

This relationship shows the same qualitative pattern for all subjects. In the early part of φ1, before the attainment of MAPmin, MAP and RRi varied consensually, so that actual BRS sequences were identified. Median BRS in the early part of φ1 was 6.6 ms·mmHg^−1^ (25°–75° centile: 5.3–11.4 ms·mmHg^−1^) in resting apnoeas in air, a value that was lower than both those observed in PRE-rest and in φ2 (p < 0.05). During resting apnoeas in oxygen, the corresponding value was 7.7 ms·mmHg^−1^ (25°–75° centile: 4.9–14.3 ms·mmHg^−1^), i.e., equal to that in air, and again significantly lower than both those observed in PRE rest (*p* < 0.05). At exercise, median BRS in the early part of φ1 was 6.4 ms·mmHg^−1^ (25°–75° centile: 3.9–13.1 ms·mmHg^−1^) in air and 6.7 ms·mmHg^−1^ (25°–75° centile: 4.1–9.5 ms·mmHg^−1^) in oxygen. These values were equal between them and did not differ significantly from the corresponding values in PRE-ex and φ2.

**Fig. 3:**
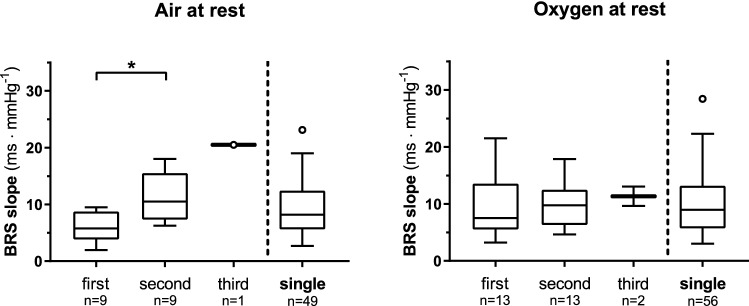
Tuckey representation of baroreflex sensitivity (BRS) computed during the φ1_up of resting apnoeas. For apnoeas characterised by multiple BRS sequences during φ1_up, the first, the second, and the third BRS are reported separately, besides the single BRS computed in apnoeas with only one BRS sequence during late φ1. *p < 0.05

After attainment of MAPmin, the pattern changed: while RRi kept decreasing, MAP started increasing. In air, this part, which did not contain BRS sequences, lasted a median of 7.2 beats (25°–75° centile: 4.9–14.6 beats) at rest, and 6.3 beats (25°–75° centile: 5.6–7.0 beats) at exercise. In oxygen, it lasted 6.3 beats (25°–75° centile: 3.2–8.1 beats) at rest, and 5.5 beats (25°–75° centile: 3.3–6.8 beats) at exercise. No differences were found among the four conditions. During φ1, the RRi attained a minimum value, after which it increased and returned close to PRE-rest values at the beginning of φ2. In air, minimal RRi was 706 ± 125 ms at rest and 598 ± 69 ms at exercise (*p* < 0.05); in oxygen minimal RRi was 698 ± 151 ms at rest and 609 ± 84 ms at exercise (*p* < 0.05). After attainment of minimal RRi, and before the end of φ1, both RRi and MAP increased consensually (φ1_up). This allowed identification of some BRS sequences: in most cases, only one BRS sequence was found (83% of the apnoeas at rest and 96% of the apnoeas at exercise), whereas multiple sequences were obtained in the remainder cases. BRS computed in φ1_up, after MAPmin, are represented in Fig. 3 for apnoeas performed at rest. Among apnoeas performed at exercise in air, only one apnoea showed two sequences (BRS was 1.25 and 11.43 ms·mmHg^−1^, respectively, for the first and the second sequence), while 40 apnoeas showed only one BRS sequence during φ1_up (median = 5.1; 25°–75° centile: 3.5–7.0 ms·mmHg^−1^). Among apnoeas performed at exercise in oxygen, two showed two sequences (1.7 and 2.9 ms·mmHg^−1^ for the first sequences, and 3.2 and 3.8 ms·mmHg^−1^ for the second sequences), while 34 apnoeas showed one single BRS sequence (median = 6.5; 25°–75° centile: 4.2–9.1 ms·mmHg^−1^).

## Discussion

In this study, we have provided the first demonstration that baroreflex resetting occurs in apnoea, namely during φ1. As is the case during the exercise transient (Bringard et al. 2017), resetting started after having attained MAPmin. Moreover, during the early part of φ1 (φ1_down), before the attainment of MAPmin, BRS was lower than in PRE-rest, but not than in PRE-ex. In analogy with what happens during exercise (Raven et al. 2006; Bringard et al. 2017), and considering that these were dry apnoeas, so that there was no or little diving reflex activation (Campbell et al. 1969; Ross and Steptoe 1980; Andersson et al. 2004), we speculate that the lower BRS in φ1_down is due to withdrawal of vagal tone.

At the beginning of apnoeas, during φ1, MAP dropped immediately for several beats (Fig. 2, φ1_down), until reaching MAPmin. Then, it restored to higher levels than baseline. RRi decreased at the beginning of apnoeas, attained a minimum some beats after attainment of MAPmin, and then recovered to reach a stable value at the beginning of φ2, similar, or slightly lower, to the prevailing RRi before apnoea. Combining the patterns of MAP and RRi on a contour plot (Fig. 2), it appears that, from the beginning of apnoea to the attainment of MAPmin, these two variables vary consensually along a baroreflex sequence. At rest, the slope of this sequence is lower than that of PRE-rest. This suggests that the MAP and RRi move toward a flatter part of the overall baroreflex curve response, farther from the centring point (maximal BRS) than in PRE rest.

An initial fall in MAP has been also described at the beginning of light exercise (Bringard et al. 2017). Nevertheless, the cause of MAP fall may differ between apnoea and exercise. The MAP fall showed at the beginning of apnoea is probably a consequence of the reduction in venous return due to the increased intrathoracic pressure linked to the high lung volumes at which apnoeas start (Korner et al. 1976; Palmero et al. 1981; Potkin et al. 2007; Breskovic et al. 2011b; Batinic et al. 2011; Fagoni et al. 2015; Stembridge et al. 2017). Differently, at the exercise onset, the MAP fall has been attributed to the sudden drop in the total peripheral resistances (Bringard et al. 2017; Fontolliet et al. 2018; Fagoni et al. 2020). The immediate pressure reduction was counteracted by an increase in HR, which was attributed to sudden vagal withdrawal at exercise onset. In agreement with this interpretation, the median BRS in φ1_down was equal in resting as in exercise apnoeas, and both were lower than the BRS in PRE-rest. The tendency toward a lower BRS in φ1_down than in PRE-ex, however, did not attain statistical significance.

Between attainment of MAPmin and attainment of a minimum RRi value, MAP and RRi varied in opposite directions, indicating baroreflex resetting. Indeed, in the majority of the volunteers, new BRS sequences were identified after few beats from MAPmin (median 5–7 beats, depending on the conditions). A similar observation was done at the exercise onset (Bringard et al. 2017), where the attainment of MAPmin preceded the baroreflex resetting. These data reinforce the concept that baroreflex resetting is not a consequence of an anticipatory central command. MAPmin was significantly higher at exercise than at rest, while MAP during PRE-ex was not different from PRE-rest, thus suggesting that the trigger of the baroreflex resetting is not the attainment of a fix value of MAPmin or MAP difference with respect to PRE-rest of PRE-ex. Further investigations need to be done to clarify baroreflex resetting mechanisms.

Between the minimum RRi and the start of φ2, MAP and RRi vary consensually again. In this phase, several apnoeas showed multiple BRS sequences. When more sequences were observed, the computed BRS increased from the minimum RRi to the beginning of φ2. This observation suggests a progressive displacement of the RRi and MAP values toward the centring point of φ2, and thus toward higher BRS.

This allows the following interpretation: (1) at the beginning of breath holding there is vagal withdrawal, as witnessed by the sudden decrease in BRS, in line with the hypothesis of Bringard et al. at exercise start (Bringard et al. 2017); (2) baroreflex resetting starts after attainment of MAPmin, so it cannot be a consequence of a central command mechanism; (3) during φ1_up, sequences occur on different baroreflex curves from those during φ1_down, due to resetting; (4) during φ1_up, BRS progressively increases to attain the BRS value of φ2, suggesting restoration of vagal drive.

This line of interpretation is coherent also with the data obtained at exercise. In fact, at exercise, during φ1_down, the BRS changes were small and non-significant, as vagal withdrawal has already occurred, at least partially, at the start of a very light exercise; yet there was baroreflex resetting also in this case, after attainment of MAPmin. Therefore, at end of φ1, the subject operated on a third baroreflex curve, which was displaced rightward, not only with respect to that for rest, but also to that for exercise. In exercise apnoeas carried out in air, there was no φ2, as expected, but in oxygen we clearly see that the OP position in φ2 is positioned to the right of that during PRE-ex (see Fig. 1). Bringard et al. (2017) postulated that, in parallel with resetting, sympathetic stimulation occurs. If this is so, considering that this study was carried out during very light exercise, 30 W, sympathetic stimulation may well determine baroreflex resetting during exercise apnoeas as well.

It is of note that this is the first study investigating BRS during both resting and exercise apnoeas. Very few studies investigated BRS during unsteady states and most of them used the Valsalva manoeuvre (Palmero et al. 1981; Grimm et al. 1998; Houtman et al. 1999), which can be compared to the early phase of apnoea. However, in those studies, the authors analysed only the phase IV of the manoeuvre, during which MAP increases, and HR decreases. We can thus speculate that those analyses were performed after the attainment of MAPmin, and probably after the resetting of baroreflex. Consequently, no direct comparison is possible between the present data and the literature data obtained during Valsalva manoeuvre.

### Methodological considerations

The BRS has been extensively measured by means of different methods and approaches which have been reviewed elsewhere (Parati et al. 2000). Unfortunately, few methods are available for the analysis of BRS during unsteady states. Among these, the sequence method (Bertinieri et al. 1988) has the advantage to be easily applied to both steady and unsteady states, thus allowing a comparison between them (Adami et al. 2013; Sivieri et al. 2015; Fagoni et al. 2015; Bringard et al. 2017).

When applying the sequence method for the computation of the BRS, either SAP or MAP has been used in literature. When applied on the same data (Akimoto et al. 2011; Bringard et al. 2017), no substantial differences have been found. Even if the sequence method as proposed by Bertinieri et al. (1988) used SAP, we consider MAP as more representative of the mean pressure stimulating baroreceptors during a heartbeat. This may be even more important during unsteady state, as is the case in this study.

Eventually, also the choice between the pulse interval (here we measured it as RRi) or its reciprocal, HR, is an open debate. Even if the sequence method (Bertinieri et al. 1988) was developed using the pulse interval, other investigators modelled the arterial baroreflex responses using HR instead. This choice is not trivial, as shown by a recent work (Taboni et al. 2018a), but, again, no studies have specifically solved this debate so far. In this article, we chose RRi, as proposed by Bertinieri et al. (1988).

Administration of oxygen during apnoeas did not modify the BRS response, nor the MAP and RRi values during the steady phases and the dynamic phase compared to air apnoeas. Moreover, data concerning the durations of the different phases between air and oxygen confirms previous findings. In resting apnoeas, φ2 is shorter in air than in oxygen, and is absent during exercise apnoeas in air, whereas it is possible to identify the φ2 during apnoeas carried out after breathing pure oxygen (Sivieri et al. 2015; Fagoni et al. 2015; Taboni et al. 2018b, 2020). Probably the higher oxygen stores compared to air apnoeas increase the total duration of apnoea. In this context, the appearance of φ2 during exercise apnoeas in oxygen allowed to analyse this steady phase, namely φ2.

## Conclusion

Apnoeas carried out at lung volumes closed to the total lung capacity allows to deepen the baroreflex analysis by means of the closed-loop method. During the first seconds of apnoeas, fast cardiovascular adaptations take place. Before attainment of MAPmin, RRi decreases following a baroreflex curve, mediated by withdrawal of vagal tone. Then, baroreflex resets in both resting and exercise apnoeas, displacing its operating point at higher MAP level that characterises the entire stable phase of breath-holding, φ2. The baroreflex resetting occurs after attainment of MAPmin, few seconds later the beginning of apnoea, suggesting that the central command does not intervene in its control. We speculate that the OP shift might be mediated via sympathetic activation.

## Abbreviations

φ1: Dynamic phase, or phase 1 of apnoea
φ1_down: Early phase 1 of apnoea (drop in arterial blood pressure)
φ1_up: Late phase 1 of apnoea (rise in arterial blood pressure)
φ2: Steady phase, or phase 2, of apnoea
BRS: Spontaneous baroreflex sensitivity
HR: Heart rate
MAP: Mean arterial pressure
MAP_min_: Minimum of MAP
OP: Operating point
PRE-ex: Steady state pre-breath holding in exercise apnoea
PRE-rest: Steady state pre-breath holding in resting apnoea
PRE-ss: Steady state pre-breath holding
RRi: Cardiac beat-to-beat interval
SAP: Systolic arterial pressure
